# Bovine serum albumin in saliva mediates grazing response in *Leymus chinensis* revealed by RNA sequencing

**DOI:** 10.1186/1471-2164-15-1126

**Published:** 2014-12-17

**Authors:** Xin Huang, Xianjun Peng, Lexin Zhang, Shuangyan Chen, Liqin Cheng, Gongshe Liu

**Affiliations:** Key Laboratory of Plant Resources, Institute of Botany, The Chinese Academy of Sciences, Beijing, People’s Republic of China; Graduate Schoo1 of the Chinese Academy of Sciences, Beijing, People’s Republic of China; ShangHai Academy of Agricultural Sciences, Forest and Fruit Tree Research Institute, Shanghai, People’s Republic of China; Heze Entry-Exit Inspection and Quarantine Bureau, Shandong, People’s Republic of China

**Keywords:** *Leymus chinensis*, Grazing, BSA deposition, RNA-seq, Apoptosis, Cell oxidative status

## Abstract

**Background:**

Sheepgrass (*Leymus chinensis*) is an important perennial forage grass across the Eurasian Steppe and is adaptable to various environmental conditions, but little is known about its molecular mechanism responding to grazing and BSA deposition. Because it has a large genome, RNA sequencing is expensive and impractical except for the next-generation sequencing (NGS) technology.

**Results:**

In this study, NGS technology was employed to characterize *de novo* the transcriptome of sheepgrass after defoliation and grazing treatments and to identify differentially expressed genes (DEGs) responding to grazing and BSA deposition. We assembled more than 47 M high-quality reads into 120,426 contigs from seven sequenced libraries. Based on the assembled transcriptome, we detected 2,002 DEGs responding to BSA deposition during grazing. Enrichment analysis of Gene ontology (GO), EuKaryotic Orthologous Groups (KOG) and Kyoto Encyclopedia of Genes and Genomes (KEGG) pathways revealed that the effects of grazing and BSA deposition involved more apoptosis and cell oxidative changes compared to defoliation. Analysis of DNA fragments, cell oxidative factors and the lengths of leaf scars after grazing provided physiological and morphological evidence that BSA deposition during grazing alters the oxidative and apoptotic status of cells.

**Conclusions:**

This research greatly enriches sheepgrass transcriptome resources and grazing-stress-related genes, helping us to better understand the molecular mechanism of grazing in sheepgrass. The grazing-stress-related genes and pathways will be a valuable resource for further gene-phenotype studies.

**Electronic supplementary material:**

The online version of this article (doi:10.1186/1471-2164-15-1126) contains supplementary material, which is available to authorized users.

## Background

Herbivore feeding is a complex process that includes wounding, defoliation, and BSA deposition [1]. Often, the leaves of a plant are completely or partially removed, affecting the photosynthetic activity, secondary metabolism, and carbohydrate relocation of plants [2–5]. Many reports have focused on stress-induced gene expression, photosynthetic capacity, root growth, and nutrient uptake after wounding and defoliation [6–9]. Resistance to herbivores depends largely upon aboveground portion of plants and on leaf-to-leaf wound signaling, which may involve electrical signaling [10]. The propagation of electrical activity leads to the expression of defense genes not only in wounded leaves but also throughout aboveground portion of plants.

Animal saliva deposited on plants (especially on leaves) is another important factor affecting plant recovery after grazing. In 1960, Vittora and Rendina first proposed that interactions between grazers and plants involve the deposition of herbivore saliva during grazing. This hypothesis has since been tested [11–14]. To date, many studies of large-herbivore BSA deposition have focused on macroscopic changes in plants, such as biomass accumulation, tiller, and increased bud initiation after grazing [15], rather than on changes in gene expression and plant physiology. On the other hand, many growth regulators in insect salivary systems have been fully researched, such as glucose oxidase, β-glucosidase [16, 17], and various growth factors in certain mammalian submaxillary glands (mainly mouse and human), including thiamine [14], nerve growth factor (NGF), transforming growth factor (TGF), and epidermal growth factor (EGF) [18], are known to have growth-regulating activity. Growth factors can intervene directly in cellular metabolism by promoting differential gene expression and are expected to be active in a variety of organisms [19]. Injecting growth-promoting substances from grasshopper saliva into *Bouteloua gracilis* stimulates tiller production [11]. Mouse and human EGF can enhance plant growth rates and promote cell division in the epicotyl [20]. However, to date, no study has reported the effects of BSA deposition by large herbivores such as cows, sheep, and camels.

Gene-expression profiling or transcriptome analysis can provide new insights to understand the molecular mechanism of grazing responses in plants. High-throughput next-generation sequencing (NGS) technologies, such as 454 (ROCHE), Solexa (Illumina), and SOLiD (ABI), have been widely and effectively used to generate large-scale transcriptome data in many plant species [21–28], including sheepgrass (*Leymus chinensis*) [29, 30]. Sheepgrass is an important forage species in the genus of *Leymus*, with good quality, high nutrition value, and various stress resistance [31–34]. Its genomic formula was N_s_N_s_X_m_X_m_ and *Elymus californicus* should be the maternal donor transferred from the genus *Elymus* to *Leymus* [35]. One copy of the haploid genome of sheepgrass contains 9.65-Gb, and high-throughput NGS technologies make it possible to generate genome resources at relatively low cost. So far, sheepgrass transcriptome databases have been generated under saline-alkaline treatment [29] and freezing treatment [30] using Roche-454 massive pyrosequencing technology. These databases provide numerous DEGs for two stresses. Recently, a comparative transcriptomics analysis of the Illumina sequencing data was conducted, and the results revealed common and distinct mechanisms for sheepgrass responses to defoliation compared to mechanical wounding [36]. Based these transcriptome databases, some grazing responsive genes were cloned and identified, such as *LcSUT1* and *LcDREB3* [37, 38].

Here, we focus on profiling the effects of herbivore saliva on sheepgrass and distinguishing BSA deposition from defoliation in grazing. In our study, we use bovine serum albumin (BSA) instead of bovine saliva to perform the grazing simulation treatment. The components of herbivore saliva are unstable and it usually contains bacterium. BSA is an important protein in bovine saliva. Its homolog has been found in ovine saliva and probably has interactions with plants [39]. In our study, in order to enrich sheepgrass transcriptome resource, accelerate our understanding of the genetic basis of grazing stress, we used Illumina GAIIx technology to sequence sheepgrass transcriptome after defoliation and grazing. We compared defoliation and grazing treatments, and identified the differentially expressed genes (DEGs) responding to BSA deposition and corresponding pathways involved in saliva effects. We performed further biochemical and morphological experiments to verify these results in transcriptome.

## Results

### Illumina sequencing and Trinity transcriptome assembly of sheepgrass

We obtained 47,782,901 raw reads from the seven libraries representing different time points or treatments via Illumina GAIIx sequencing (Table 1). The sequence reads generated in this study were deposited in the NCBI sequence-read archive (SRA065691). The raw reads were filtered by a stringent criterion as described in Methods. The remaining reads were considered clean. An additional file show this in more detail (see Additional file 1).

**Table 1:** **Statistics summary of Illumina sequencing data generated for sheepgrass transcriptome**

Library	Time point ^1^	Treatment ^2^	Raw reads ^3^	Clean reads ^4^	Average length (bp) ^5^	454 mapping ^6^
**C**	---	Control	6,260,824	5,912,096	95	1,996,649
**D2**	2 h	Defoliation	9,072,621	8,514,655	93	1,801,714
**D6**	6 h	Defoliation	9,706,319	9,087,056	93	2,200,569
**D24**	24 h	Defoliation	7,369,430	6,917,684	92	2,265,088
**G2**	2 h	Grazing	5,117,612	4,732,256	92	1,721,681
**G6**	6 h	Grazing	7,593,520	7,088,551	95	2,457,870
**G24**	24 h	Grazing	2,662,575	2,429,333	95	858,130

^1^The time point after corresponding treatments.

^2^The type of the treatment in sheepgrass.

^3^Total number of reads separated from each library.

^4^Number of high-quality reads corresponding to mRNA sequences used for further analysis.

^5^Average length of high-quality clean reads.

^6^Number of reads mapped to sheepgrass 454 transcriptome sequencing dataset [29].

We obtained 120,426 contigs (≥200 bp) using the Trinity assembly software. The mean contig size was 634 bp, and the contig size ranged from 201 to 28,343 bp. About one-third of the contigs were longer than 500 bp, and 20,816 contigs were longer than 1,000 bp. Additional file 2 show the quality of the assembly transcripts in more detail (see Additional file 2).

### Contig assembly and gene overview

**Figure 1:**
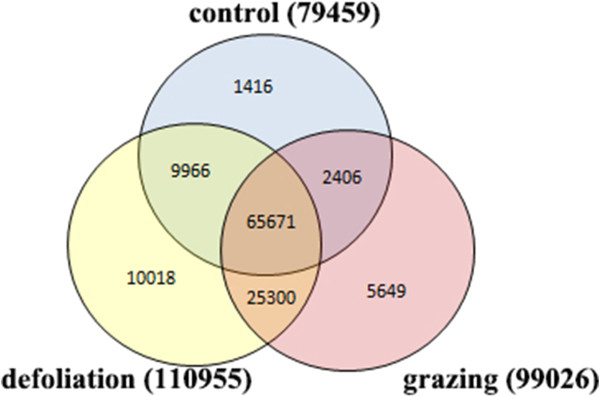
**The venn diagram of gene counts in the control, defoliation and grazing treatments.** The control contains Library C. The defoliation treatment contains Library D2, D6, and D24. The grazing treatment contains Library G2, G6, and G24. Numbers in parentheses are total number of expressed genes in each treatment.

Using the Trinity *de novo* assembly program, a total of 120,426 contigs were obtained. 79,459 contigs were detected in Library C (the control). 83,189, 85,184 and 77,786 contigs were detected in three defoliation libraries (D2, D6, and D24), respectively. Excluding these repeated contigs in the three samples, there were 110,955 contigs detected in defoliation libraries. 69,112, 80,829 and 55,874 contigs were detected in three grazing libraries (G2, G6, and G24), respectively, with a total of 99,026 in grazing libraries. The three treatments are summarized in Figure 1. The quality of the contigs in the seven samples is shown in Additional files 3 & 4.

### Functional annotation and descriptive profile

**Figure 2:**
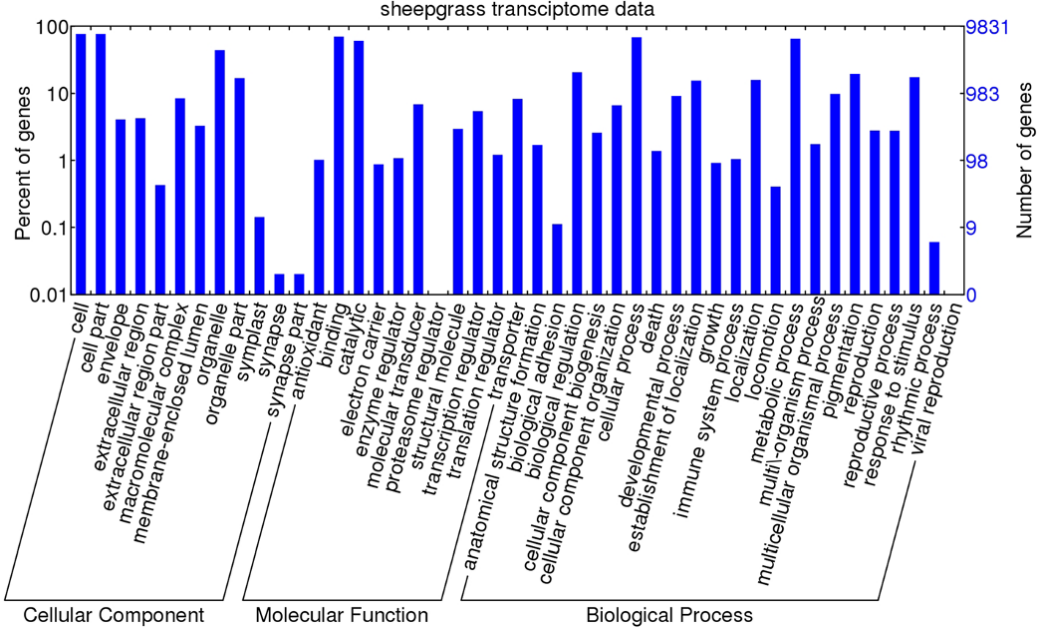
**GO classifications of assembled transcripts using WEGO software (** http://wego.genomics.org.cn **).** The genes were assigned to three main categories: biological process, molecular function and cellular component. The right hand y-axis indicates the number of annotated genes. The left hand y-axis indicates the percentage of annotated genes.

Gene ontology (GO) assignments were used to classify the functions of the predicted sheepgrass genes expressed in response to grazing stress. Based on sequence homology, 9,831 genes were assigned at least one GO term, including 49 second-level functional categories (Figure 2). An additional docx file show the summary of WEGO output data in more detail (see Additional file 5). Among the assigned terms, “cell” (7,508 terms, 76.4%), “cell part” (7,508 terms, 76.4%), “organelle” (4,311 terms, 43.9%) and “organelle part” (1,643 terms, 16.7%) were dominant in the cellular component. “Cellular process” (6,690 terms, 68.1%), “metabolic process” (6,378 terms, 64.9%), “biological regulation” (2,011 terms, 20.5%), “pigmentation” (1,902 terms, 19.3%), and “response to stimulus” (1,701 terms, 17.3%) were dominant among biological processes. The absolute majority of molecular-function terms were clustered in “binding” (6,850 terms, 69.7%) and “catalytic activity” (5,927 terms, 60.3%).

**Figure 3:**
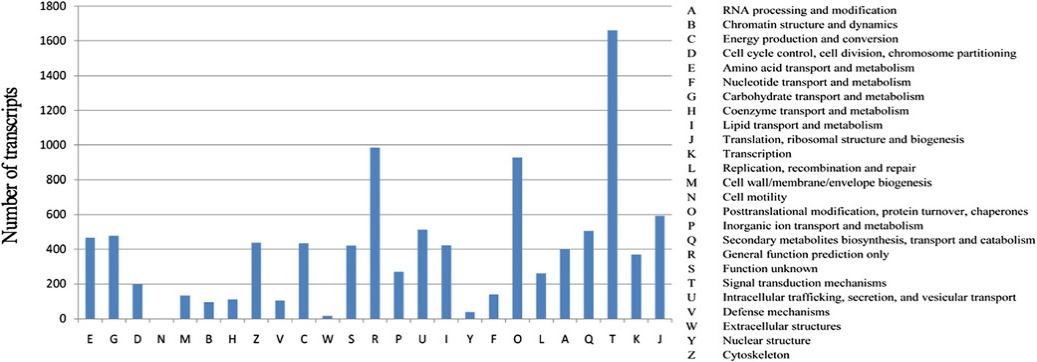
**KOG function classifications of assembled transcripts.** The contigs were assigned to the KOG database to predict possible functions. A total of 9,985 contigs were assigned to 25 categories.

To further evaluate the completeness of the *de novo* transcriptome assembly and to predict the gene functions, all assembled transcripts were compared against the EuKaryotic Orthologous Groups (KOG) database. This comparison revealed 9,985 sequences with significant homology, each of which was assigned to the appropriate KOG cluster. These KOG classifications were grouped into 25 functional categories (Figure 3). The five largest categories were “signal-transduction mechanisms” (16.64%), “general function prediction only” (9.87%), “posttranslational modification, protein turnover, chaperones” (9.29%), “translation, ribosomal structure, and biogenesis” (5.34%), and “intracellular trafficking, secretion and catabolism” (5.14%).

The Kyoto Encyclopedia of Genes and Genomes (KEGG) is a database resource for the systematic understanding of high-level gene functions in terms of biological networks, such as the cell, organism, and ecosystem, from molecular-level information (http://www.genome.jp/kegg/). The assembled transcripts were searched against the KEGG database using BLASTX with a cut-off E-value of 10^−5^ to identify the biological pathways related to grazing responses in sheepgrass. We obtained 6,820 matching terms, which were assigned to 275 KEGG pathways in 5 main biological processes. The major pathways were “biosynthesis of amino acids” (ko01230, 191 transcripts), “ribosome” (ko03010, 165 transcripts), “carbon metabolism” (ko01200, 163 transcripts), “purine metabolism” (ko00230, 137 transcripts), “spliceosome” (ko03040, 119 transcripts), “protein processing in endoplasmic reticulum” (ko04141, 118 transcripts), and “RNA transport” (ko03013, 114 transcripts).

We applied gene enrichment analysis between the grazing libraries (G2, G6, and G24) and the control (C) in Table 2. The results showed 10 GO second-level functional categories responded to grazing treatment. These groups were “transporter activity”, “oxidoreductase activity”, “catalytic activity” and “lyase activity” of molecular function, “amino acid and derivative metabolism”, “transport” and other three groups of biological process, “external encapsulating structure” of cellular component. In KOG functional classification, “Lipid transport and metabolism”, “Amino acid transport and metabolism” and “Energy production and conversion” were significantly different (q-value < 0.01). 10 KEGG pathways showed significant difference (q-value < 0.01) between the grazing treatment and the control. Pathways “Two-component system” and “ABC transporters” were two of the most different ones.

**Table 2:** **Gene enrichment analysis of DEGs from grazing vs. control**

GO second-level functional groups (DEG number 591; background number 9,831)	q-value
Transporter activity	4.54E-12
Oxidoreductase activity	1.17E-06
Catalytic activity	3.02E-06
Amino acid and derivative metabolism	3.49E-05
Transport	0.000226
Lyase activity	0.000369
Metabolism	0.000499
Pathogenesis	0.000773
**KOG categories (DEG number 337; background number 9,985)**	**q-value**
Carbohydrate transport and metabolism	0.000494
Lipid transport and metabolism	0.002497
Secondary metabolites biosynthesis, transport and catabolism	0.002497
Energy production and conversion	0.002974
**KEGG pathway ID (DEG number 420; background number 6,820)**	**q-value**
02020 Two-component system	4.69E-22
02010 ABC transporters	1.20E-19
00281 Geraniol degradation	0.002774
00640 Propanoate metabolism	0.005709
00330 Arginine and proline metabolism	0.005841
00500 Starch and sucrose metabolism	0.005841
00930 Caprolactam degradation	0.005841
00071 Fatty acid degradation	0.006474
00380 Tryptophan metabolism	0.006716
00250 Alanine, aspartate and glutamate metabolism	0.008322

### Differentially expressed genes (DEGs) in response to BSA deposition

**Figure 4:**
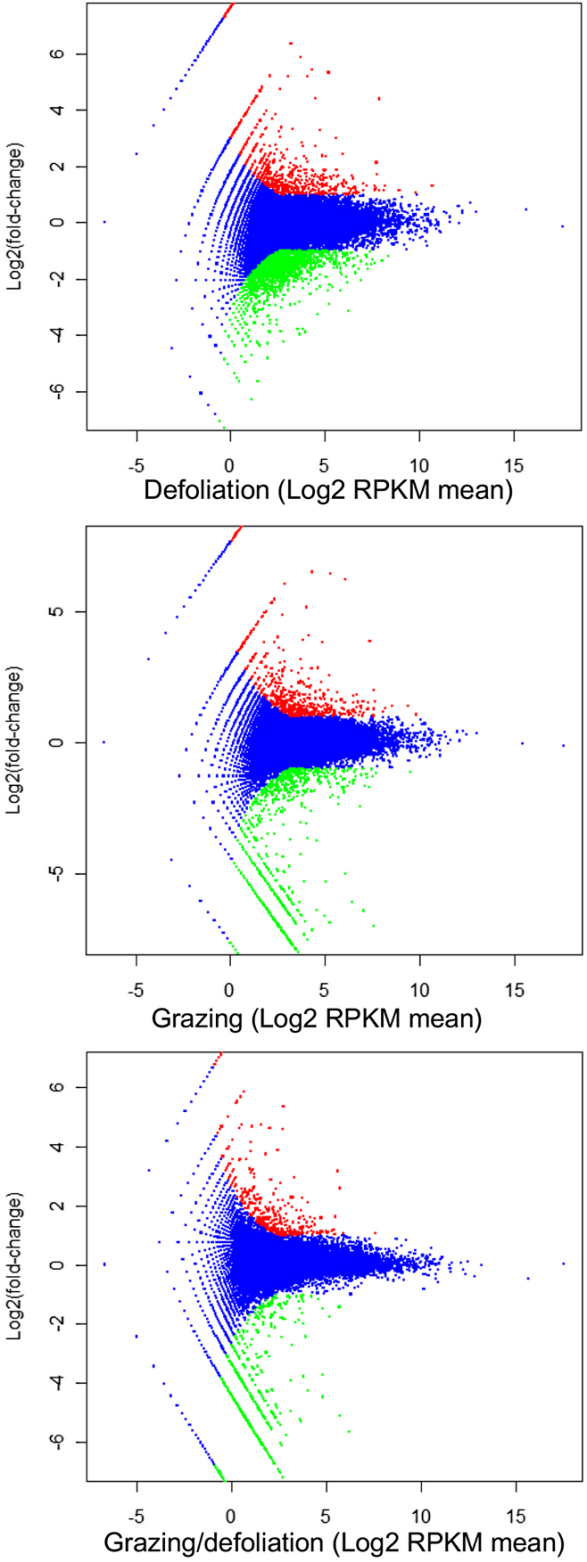
**The expression change of DEGs responded to defoliation, grazing, and BSA deposition in MA plots.** The MA plot uses M as the y-axis (log2 fold-change) and A as the x-axis (log2 RPKM mean). The red dots stand for the DEGs which are up-regulated, and the green dots stand for those down-regulated DEGs.

To investigate the changes in gene expression and understand the critical genes involved in the response of sheepgrass to BSA deposition, we collected the RPKM means from the grazing (G2, G6, and G24) and defoliation libraries (D2, D6, and D24) and analyzed the significant difference genes using *DEGseq* package [40]. The differentially expressed genes (DEGs) in response to defoliation (D), grazing (G) and BSA deposition (G vs. D) were determined from the changes in expression of defoliation vs. control, grazing vs. control, and grazing vs. defoliation. Using a screening criterion of FDR < 0.001, we found 2,002 genes that responded to BSA deposition, of which 365 were induced and 1,637 were inhibited. We found 3,156 genes that responded to grazing and 2,759 genes that responded to defoliation (Table 3). Figure 4 show the details of expression changes of DEGs in MA plot. A cluster analysis of the libraries of control, defoliation, and grazing was performed using the heat map shown in Additional file 6.

**Table 3:** **Gene statistics of the differentially expressed genes**

Type of the differentially expressed genes	Up-regulated genes (p < 0.001)	Down-regulated genes (p < 0.001)	Total number
Defoliation (D vs. C)	647	2,112	2,759
Grazing (G vs. C)	592	2,564	3,156
BSA deposition (G vs. D)	365	1,637	2,002

**Figure 5:**
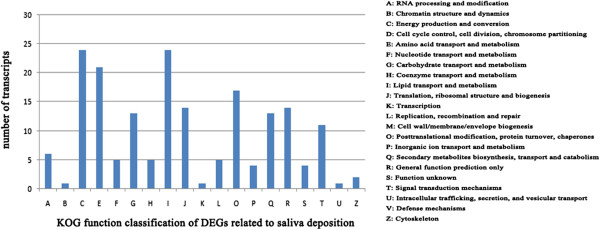
**KOG function classifications of DEGs responding to BSA deposition.** The DEGs were aligned to the KOG database to predict possible functions related to BSA deposition. A total of 185 DEGs were assigned to 19 categories.

GO functional-enrichment analysis was performed for 377 GO terms from BSA deposition (G vs. D) DEGs compared to the 9,831 GO terms from the full transcriptome at a Bonferroni-corrected *P*-value ≤ 0.05. The results showed the biological-process categories “cell death” and “transport” and the molecular-function categories “antioxidant activity”, “oxidoreductase activity”, and “transporter activity” were significantly enriched (Table 4). In the KOG enrichment analysis, 185 KOG annotation terms were assigned to the saliva-deposition DEGs. “Energy production and conversion”, “amino acid transport and metabolism” and “lipid transport and metabolism” were the significant enrichment categories (Figure 5, Table 5).

**Table 4:** **Enriched GO categories of DEGs from BSA deposition (G vs. D)**

GO categories (DEG number 377; background number 9,831)	q-value
Transporter activity	9.12E-12
Oxidoreductase activity	1.57E-06
Catalytic activity	4.57E-06
Amino acid and derivative metabolism	5.50E-06
Transport	0.000254
Lyase activity	0.000446
Metabolism	0.000641
Pathogenesis	0.001039
External encapsulating structure	0.001629
Integrase activity	0.006320
Ligase activity	0.007898
Biosynthesis	0.008923
Catabolism	0.008923

**Table 5:** **Enriched KOG categories of DEGs from BSA deposition (G vs. D)**

KOG categories (DEG number 185; background number 9,985)	q-value
Energy production and conversion	5.06E-06
Lipid transport and metabolism	5.06E-06
Amino acid transport and metabolism	0.000436

In the KEGG pathway enrichment analysis, 307 annotation terms were obtained for the saliva-deposition DEGs. These 307 terms belonged to 136 pathways. We found that 11 pathways were statistically enriched in the DEGs responding to BSA deposition. As shown in Table 6, the most enriched pathways were related to environmental stress responses. For example, two-component systems play important roles in signal transduction in response to environmental stimuli and growth regulators, light and osmotic stress.

**Table 6:** **Enriched pathways of DEGs from BSA deposition (G vs. D)**

Pathway ID (total DEG number 307; background number 6,820)	DEGs number	q-value
**02020 Two-component system**	40	7.04E-19
**02010 ABC transporters**	35	5.83E-16
**00380 Tryptophan metabolism**	12	0.002054
**00281 Geraniol degradation**	7	0.002128
**00780 Biotin metabolism**	6	0.008817

By aligning our reads to the reference transcriptome dataset obtained by Roche-454 massive pyrosequencing technology [30], we obtained further details and annotations (Table 1). Among the DEGs responding to BSA deposition were serine/threonine protein kinase, apoptotic ATPase, and aldehyde dehydrogenase-family proteins. Most of these genes showed different modes of expression 24 h after defoliation or grazing, as shown in Table 7.

**Table 7:** **Cell oxidative and apoptosis related genes in DEGs**

Gene ID	Annotation	Expression ^a^
Contig24413	Apoptosis-promoting RNA-binding protein TIA-1/TIAR	
Contig23357	Serine/threonine protein kinase	
Contig14090	Molecular chaperones mortalin /PBP74 /GRP75, HSP70 superfamily	
Contig24049	Metacaspase involved in regulation of apoptosis	
Contig08269	Apoptotic ATPase	
Contig07414	Apoptotic ATPase	
Contig11179	Cullin-1	
Contig03643	Aldehyde dehydrogenase family 2 member B4	
Contig25560	Exosome complex exonuclease rrp40	
Contig25022	Cytosolic Ca^2+^-dependent cysteine protease (calpain), large subunit (EF-Hand protein superfamily)	
Contig10432	L-ascorbate peroxidase	
Contig14733	Catalase	
Contig18439	Glutathione peroxidase	
Contig26462	Ferritin	
Contig26780	Ferric reductase, NADH/NADPH oxidase and related proteins	
Contig23291	Thioredoxin	
Contig08847	Glutaredoxin and related proteins	

“^**a**^” Relative expression abundance of genes after sheepgrass treated by defoliation and grazing at 2 h, 6 h, and 24 h (relative expression abundance of genes in the control =1). The values which are statistically significantly changed (an FDR(false discovery rate) < 0.001) in comparison with control are marked by asterisks (*). The black bars stand for the gene expression levels in defoliation treatments, and the red bars stand for the gene expression levels in grazing treatments.

### Programmed cell death after clipping and the effect of BSA

**Figure 6:**
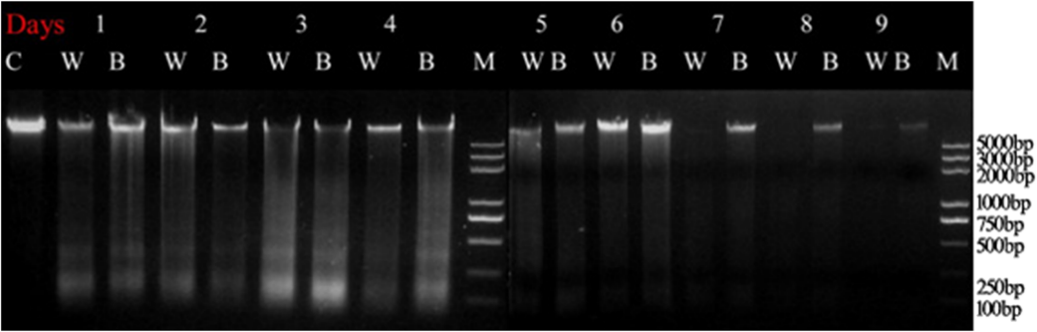
**Agarose gel images of the DNA fragments.** M refers to the DNA marker 2000, C refers to the control, W refers to the water-treated leaf samples, and B refers to the BSA-treated leaf samples. Lanes from left to right: control, water-treated and BSA-treated leaf samples from days 1 to 9 post-treatment.

To confirm the reliability of apoptosis in response to BSA deposition during grazing, DNA fragments from cells from the cut ends of leaves treated with water or BSA were analyzed using agarose-gel electrophoresis. As shown in Figure 6, most of the DNA fragments appeared on the third and fourth days after clipping in both the BSA- and water-treated groups. Genomic DNA declined by day 5 and disappeared by day 7 in the water-treated group. In the BSA-treated group, however, genomic DNA decreased slowly and was still observed on day 9.

### Accumulation of oxidative-stress-related factors in grazed sheepgrass

**Figure 7:**
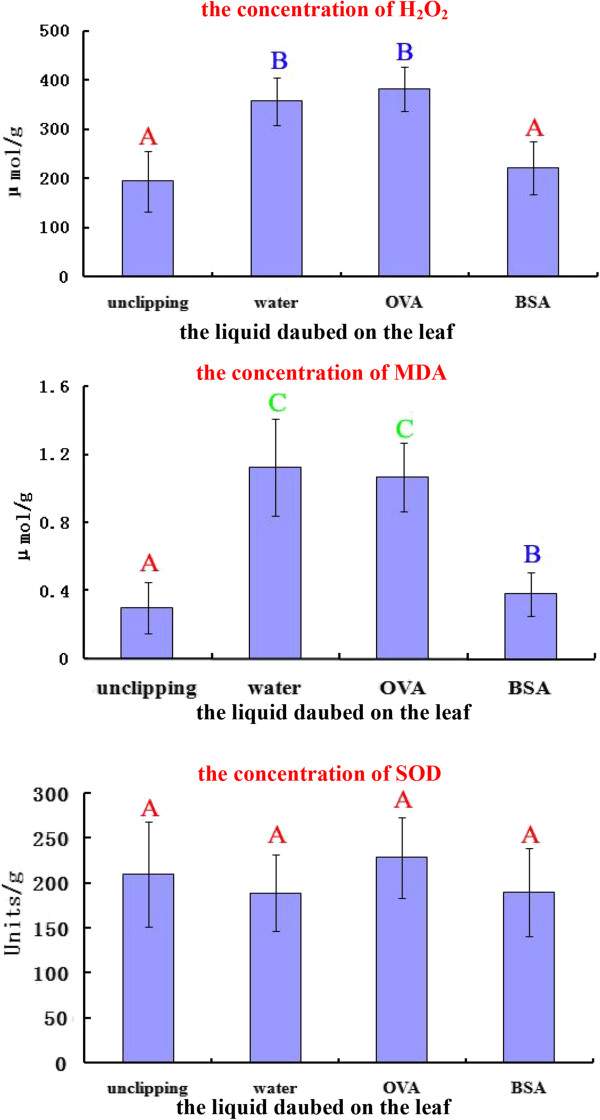
**The concentration analysis of H2O2, MDA, and SOD in the leaf scar cells.** The statistical data were treated by ANOVA (Analysis of Variance) test and S-N-K (Student-Newman-Keuls) test. “H_2_O_2_” and “MDA” groups were significant while “SOD” group was not significant by ANOVA test. The same letter over two treatments stands for no significant difference and two treatments under different letters were significantly different in S-N-K test.

The H_2_O_2_ levels increased significantly in the water- and ovalbumin (OVA)-treated groups compared to the unclipped controls (*p* < 0.01). The OVA treatment was used as a control for BSA to eliminate the interference of proteins daubed on the cut surface. Daubing with BSA marginally affected the H_2_O_2_ levels in the cells, but this difference was not significant compared to the controls (Figure 7).

The changes in malondialdehyde (MDA) were similar to those in hydrogen dioxide (H_2_O_2_). The MDA levels in the water-treated and OVA-treated group were approximately 3- to 4-fold higher than those in the unclipped and BSA-treated groups, and this difference was significant at *p* < 0.01. The MDA levels in the BSA-treated cells increased slightly compared to the controls (Figure 7).

The superoxide dismutase (SOD) levels in the treated groups did not differ significantly from those of the controls (Figure 7). Thus, the grazing treatment using BSA affected the oxidative-stress-related factors in sheepgrass only slightly.

### Analysis of leaf-scar lengths

**Figure 8:**
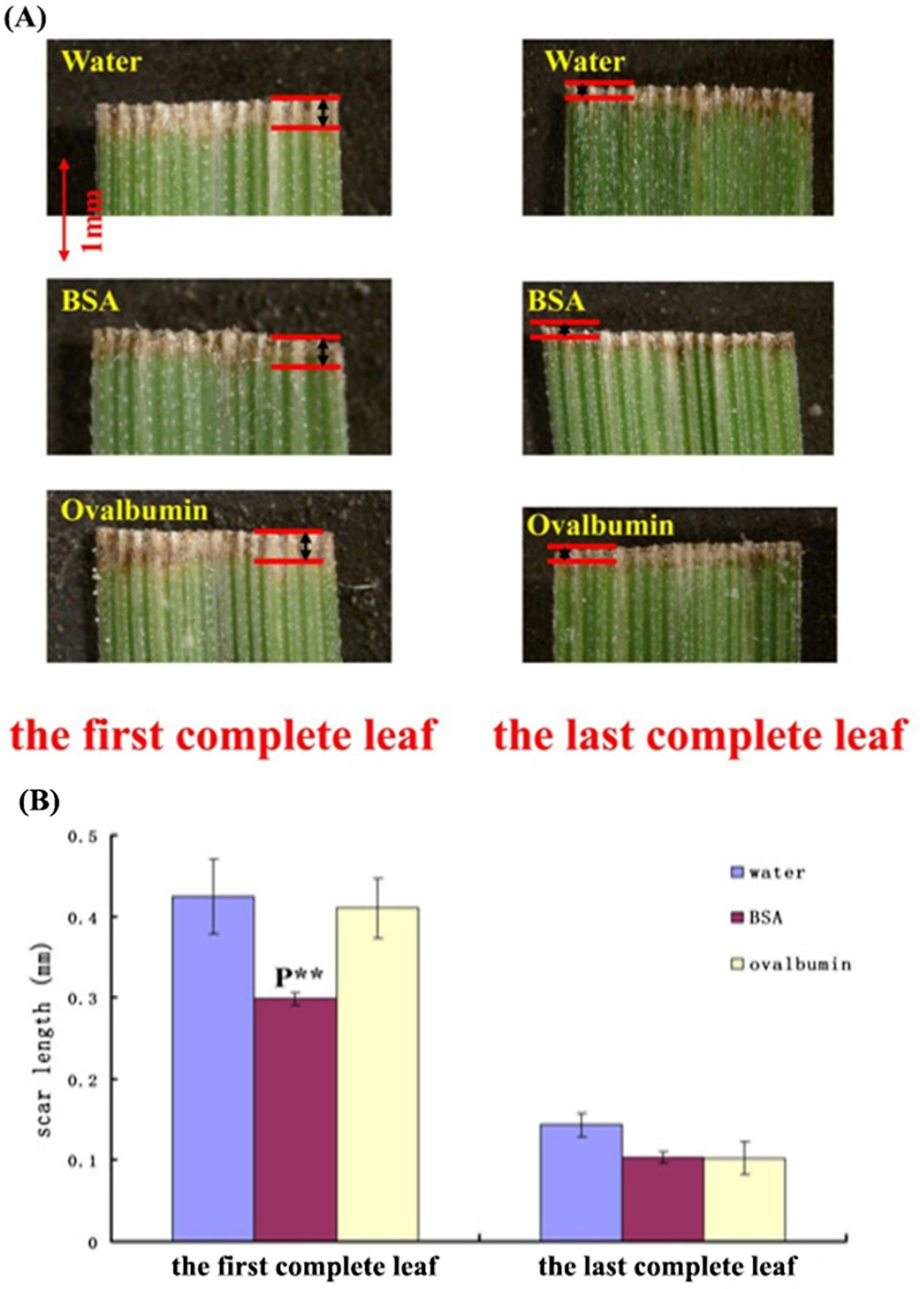
**Analysis of the leaf scars. (A)** Images of the leaf scar measurements in the first and last complete leaves. A red line showing the position of leaf scars. **(B)** Statistical analyses. Left: data collected from the first complete leaves. Right: data collected from the last complete leaves. P** refers to a significant difference (p < 0.01) between the BSA and water treatments.

The lengths of the clipping scars on the leaves reflect changes in the sheepgrass transcriptome at the macroscopic level, especially changes in the cell oxidative status and apoptosis. For the first completely expanded leaf, the scars on the leaves daubed with water were approximately 1.3-fold longer than those on the leaves daubed with BSA (Figure 8), and this difference was statistically significant (*p* < 0.01). The phenotype of the OVA-treated leaves was similar to that of the water-treated leaves but markedly different from that of the BSA-treated leaves. However, the leaf-scar length on the last completely expanded leaf was similar in all treatments.

## Discussion

Grazing is an important and frequent stress for pasture and prairie plants. Plant scientists have long studied the effects of grazing on plants as a single process. However, grazing is a complex process that involves wounding effects caused by herbivore feeding, defoliation effects due to leaf-surface loss during grazing, and the deposition of herbivore saliva onto the surface of plants. Some studies have reported that wounding can stimulate plant growth but clearly differs from grazing [17, 41–43]. Defoliation affects root development in grasses [44, 45] and alters the carbohydrate-metabolism pathway in rice (unpublished). On the other hand, scientists have examined how plants respond to BSA deposition for decades [11, 12]. Saliva has been found to stimulate plant growth, enhance tiller and increase biomass [14]. However little is known about the molecular mechanisms of grazing responses and the genetic and functional differences among three components of grazing. To investigate the genetic profile of the grazing response in sheepgrass and to elucidate the differences in mechanism between the saliva-deposition response and the responses to other grazing components, we analyzed the transcriptomes of control, defoliated, and grazed plants using RNA-seq.

### Sequence quality and annotation

Illumina RNA-seq technology had been widely used in genome-wide analyses of cotton (*Gossypium hirsutum*), radish (*Raphanus sativus*), *Brassica juncea*, and *Brassica pekinensis* [46–49]. Here, we obtained more than five million raw reads from most samples. Using the Trinity transcriptome-assembly software, a total of 120,426 assembled transcripts were obtained from seven sample libraries (untreated control, defoliation, and simulated grazing). Of these transcripts, 14,240 were annotated by BLASTX and functional-bioinformatics analyses, including the GO, KOG, and KEGG databases. No genome of sheepgrass or close relative species was available, so most of our transcripts cannot hit known proteins. In addition, a relative stringent blast parameter (E-value < 1e-5) might discard a part of known hits. We obtained 74,087 GO terms, 9,985 KOG terms and 7,240 KEGG pathway terms for all transcripts combined. The gene-transcription profiles of sheepgrass after grazing were stored in an annotated-gene catalog to provide a molecular understanding of grazing responses. The remaining un-annotated transcripts may represent a sheepgrass-specific gene pool. These results provide a solid foundation for further studies of the molecular mechanisms of grazing responses and for identifying grazing-related genes in this species.

### Gene enrichment analysis and the effects of grazing

Grasslands and grass re-growth after grazing are very important for both the ecosystem and human dairy food supply on this planet. Grazing is a processing that have multiple components including at least wound, defoliation, and BSA deposition. Grazing often removes completely or partially the leaf part of plants. After grazing, plants have to transport the carbohydrate such as sucrose and other energy substance due to root sink demand [50–52]. In our enrichment analysis (Table 2), “transporter activity” and “amino acid and derivative metabolism” in GO categories, “Amino acid transport and metabolism” and “Lipid transport and metabolism” in KOG categories, and KEGG amino acid metabolism pathways were significant enrichment in grazing treatment. The results indicated that amino acid metabolism involved in plant grazing response. The amino acid metabolism pathways may contribute to protein biosynthesis and plant recovery after grazing, and the related genes are worth further study.

### Differential genetic profiles in response to BSA deposition

Sheepgrass is a dominant grassland species in northeastern China and Inner Mongolia and is known for its adaptability to grazing and excellent forage quality among perennial grasses [53]. In this study, 2,759 genes were expressed differently between the control and defoliation libraries, indicating that these genes responded to defoliation. Similarly, 3,156 genes were expressed differently between the control and grazing libraries, indicating that these genes responded to the combined effects of defoliation and BSA deposition. Furthermore, 2,002 genes were expressed differently between the defoliation and grazing libraries, indicating that these genes responded to BSA deposition. The only difference between these two treatments was the liquid deposited on leaves.

As shown in Table 3, most of the DEGs were down-regulated. In the KOG enrichment analysis (Table 5), the down-regulated DEGs were enriched in lipid transport and metabolism; energy production and conversion; and amino-acid transport and metabolism. Thus, several functionally linked metabolic pathways were down-regulated in response to grazing. This result is consistent with a proteomic analysis of rice after ovine BSA deposition [54], in which the authors found that most photosynthesis-related, energy-related, and carbohydrate-metabolism related proteins were down-regulated. BSA deposition on plants is accompanied by a multitude of stresses including oxidative stress, pathogenesis, and wounding [55–57].

We examined transcript expression in sheepgrass at three time points following grazing. The gene-expression analysis helped to clarify how the expression of the DEGs adjusts to grazing stress. When G2 (2 h after treatment), G6 (6 h after treatment), and G24 (24 h after treatment) were compared to C (no treatment), 2,367 genes were differentially expressed after 2 h, 2,285 genes were differentially expressed after 6 h, and 1,692 genes were differentially expressed after 24 h. Among these, 1,074 genes were differentially expressed at all three time points, indicating that about half of the DEGs were stable during the first 24 h after grazing. The key pathways involved in grazing-response mechanisms may contain these genes.

### Apoptosis-related DEGs

In plants, apoptosis is induced by multiple stresses, including salt, nitric oxide, oxidative stress, and wounding [58–61]. This study also shows apoptosis-related DEGs in response to grazing. Based on functional-enrichment analysis, the apoptosis pathway is significantly involved in the saliva-deposition response. In the DNA-fragmentation experiment, we found fewer DNA fragments and delayed DNA fragmentation following BSA deposition compared to defoliation.

The apoptosis-promoting RNA-binding proteins TIA-1 and TIAR (RRM superfamily) was detected among the DEGs. These proteins promote DNA fragmentation in digitonin-permeabilized thymocytes and are pro-apoptotic factors that influence some aspect of RNA metabolism [62]. In our expression-mode analysis, TIA-1/TIAR was significantly down–regulated in the grazing treatment compared to the control and defoliation treatments. Correspondingly, less DNA ladder was seen after BSA deposition.

The expression of the serine/threonine kinase PAK4 increases the phosphorylation of the pro-apoptotic protein BAD and inhibits the activation of caspase, which protects cells against apoptosis [63]. Hsp70 and many other heat-shock proteins can overcome both caspase-dependent and caspase-independent apoptotic stimuli and confer immortality in various cell types [64]. Metacaspases are evolutionarily distant caspase homologs that are found outside the Metazoa and are known to play key roles in programmed cell death (PCD) [65]. However, whether metacaspases in plants function as caspases is controversial [66]. These apoptosis-inhibiting genes were all up-regulated only in the grazing treatment.

ATPases, including Na^+^, K^+^, and H^+^ ATPases, play critical roles in apoptosis [67, 68]. Apoptotic stimuli impair Na^+^- and K^+^-ATPase activity as a mechanism of neuronal death mediated by concurrent ATP deficiency and oxidative stress [69]. Several genes were annotated as “apoptotic ATPases” in the KOG functional analysis and their expression modes are shown in Table 7.

Additional apoptosis- or PCD-related genes were detected among the DEGs. Cullin controls non-lysosomal-mediated protein degradation and thus cell death [70]. Aldehyde dehydrogenase-2 (ALDH2) converts acetaldehyde into acetate, and over-expression of an ALDH2 transgene prevents acetaldehyde-induced cell injury and apoptosis [71]. The exosome-complex exonuclease rrp40 forms part of the exosome, which is important to the RNA-processing machinery of eukaryotes and functions in RNA degradation in both the nucleus and the cytoplasm [72]. A Ca^2+^-dependent cysteine protease (CDP) is associated with anoxia-induced root-tip death in maize [73].

Programmed cell death or apoptosis is an integral part of plant ontogenesis and plays a fundamental role in plant development. According to the above described genes, we suggest that there are some important pathways of apoptosis, in response to BSA deposition during grazing in plants.

### Oxidative-stress-related DEGs

Cellular oxidative stress is a common challenge for plants that usually accompanies wounding [74] or senescence [75] and is closely associated with apoptosis [76]. Many apoptosis-inducing agents are either oxidants or stimulators of cellular oxidative metabolism. In our measurements of cellular oxidative status, H_2_O_2_, and MDA but not SOD increased substantially after grazing. However, cells from sheepgrass leaves subjected to BSA deposition showed significantly low H_2_O_2_ and MDA levels. In addition, we detected cellular oxidative-control genes among the DEGs.

Major ROS-scavenging enzymes in plants include superoxide dismutase (SOD), ascorbate peroxidase (APX), catalase (CAT), glutathione peroxidase (GPX), and peroxiredoxin (PrxR) [77]. We found no SOD genes among the DEGs, indicating that SOD expression was stable following the grazing process. This finding is consistent with the cellular oxidative-status experiment. Furthermore, we found no PrxR DEGs. However, the DEGs included APX, CAT, and GPX genes (Table 7). These three ROS-scavenging enzymes were up-regulated in the grazing treatment compared to the control. Their expression was also relatively higher in the grazing treatment than in the defoliation treatment, possibly explaining the low H_2_O_2_ and MDA levels after BSA deposition.

The DEGs included other cellular oxidative-stress-related genes. Ferritin prevents the formation of the highly toxic HO·radical via the metal-dependent Haber–Weiss reaction or the Fenton reaction [78]. Several studies have shown that biotic and abiotic stresses are accompanied by an oxidative burst mediated by NADPH oxidases [79, 80]. The glutaredoxin (Grx) and thioredoxin (Trx) pathways use NADPH to reduce the disulfide bonds that form in some cytoplasmic enzymes during catalysis. The thioredoxin system consists of thioredoxin reductase and thioredoxin, and the glutaredoxin system is composed of glutathione reductase, glutathione, and three glutaredoxins [81]. The differential expression of these genes suggests that grazing stress or BSA deposition was closely related to cellular oxidative changes.

**Figure 9:**
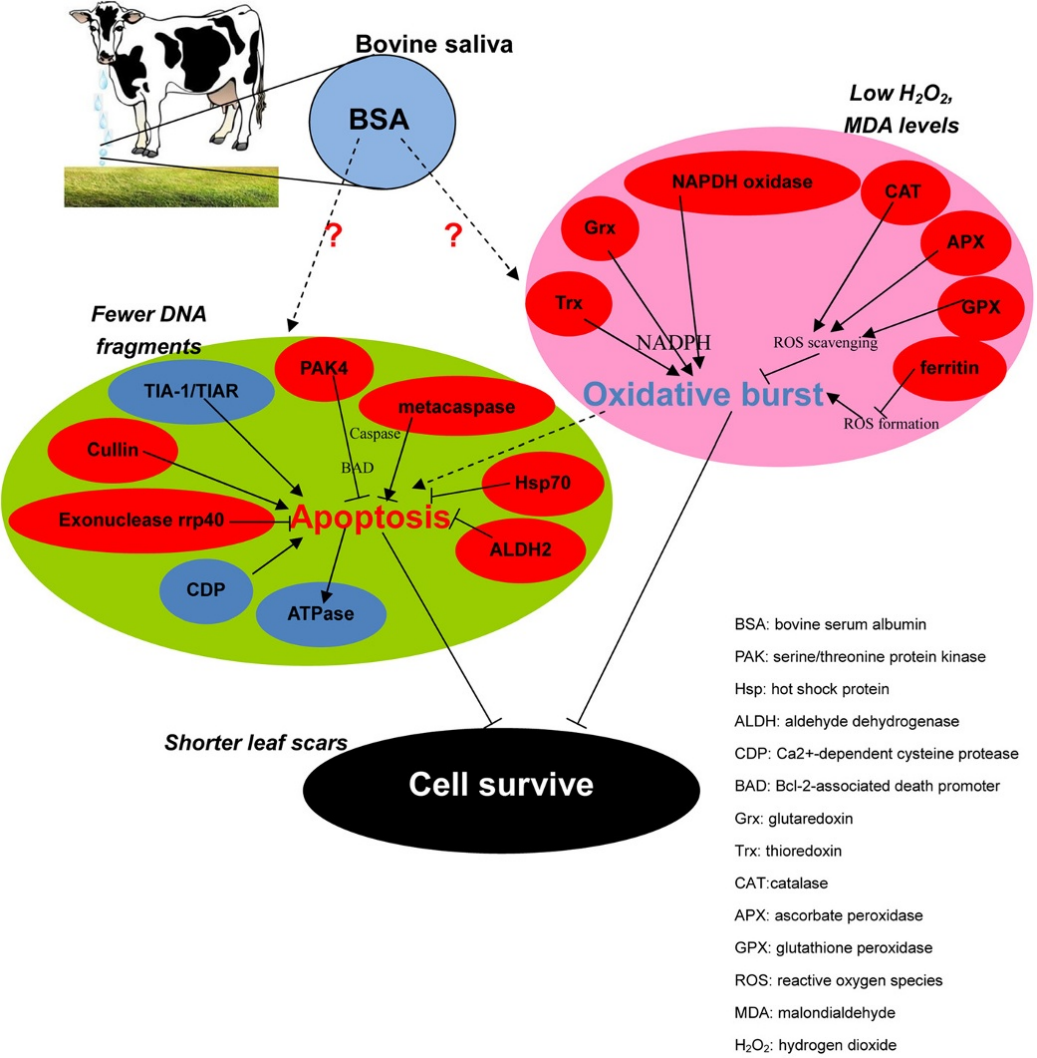
**Molecular mechanism of sheepgrass responds to BSA deposition.** The DEGs in red oval frames are up-regulated and those in blue oval frames are down-regulated in expression levels in grazing libraries compared with defoliation libraries. The arrow lines stand for the effect of activation. The blunt lines stand for the effects of inhibition. The dotted lines stand for the unknown effects.

The present transcriptome-sequencing results and related biochemical experiments help to elucidate the response of sheepgrass to BSA deposition. After grazing, the plants receive the signal of BSA deposited on the leaves and elevate the expression of ROS-scavenging enzymes and antioxidant pathways to respond to the subsequent oxidative burst in the cells. The decreased cellular oxidative levels result in fewer apoptotic cells in the grazing wound, accelerating the recovery from the grazing stress in plants. A model of sheepgrass responded to BSA deposition was indicated in Figure 9. The co-evolution of plants and herbivores in grazing systems may have led to this antioxidant mechanism in response to BSA deposition.

## Conclusion

To investigate the molecular mechanism of grazing responses, we performed transcriptome sequencing and analysis to identify DEGs in sheepgrass subjected to simulated BSA deposition. Our results show that BSA deposition triggers differential gene expression compared to defoliation and other grazing components. Based on a functional analysis of the saliva-deposition DEGs, the cellular-antioxidant and apoptotic pathways apparently respond to grazing stress. Macroscopic changes confirm the effects of these two pathways in sheepgrass. Although the connection between the two pathways requires further evidence, we believe that the saliva-deposition-induced pathways work together to contribute to plant recovery after grazing.

## Methods

### Plant materials, growth conditions, and treatments

All sheepgrass plants (Zhongke No. 3) were obtained from the field 4 weeks before the initiation of the experiment. Seedlings in good condition were collected and transplanted into trays in the greenhouse. The trays were filled with a mixture of vermiculite and commercial potting soil at a ratio of 1:2. The plates were placed in a greenhouse at 25°C and 70% relative humidity. The aboveground portion of each plant was cut off, and plants were allowed to re-grow to the 5- or 6-leaf stage. To initiate the experimental treatments, two-thirds of the aboveground portion of each plant was cut off. For the defoliation treatment, water was then daubed on the cut ends of the leaves. For the grazing treatment, a BSA solution (1 mM) was daubed on the cut ends of the leaves. The remaining aboveground portions of the plants were collected 2, 6, and 24 h after the clipping and daubing treatments. The corresponding parts of the control (unclipped) seedlings were collected at the same time. All harvested seedlings of each treatment were immediately frozen in liquid nitrogen and stored at −80°C. The clipping and daubing treatments were conducted at 10:00 AM and all the materials collection was conducted in daylight hours to reduce the effort of circadian rhythmicity. In total, seven samples were obtained: C (control); D2, D6, and D24 (2, 6, and 24 h after defoliation, respectively); and G2, G6, and G24 (2, 6, and 24 h after grazing, respectively).

### RNA-seq library preparation and Illumina sequencing

Total RNA was extracted using the TRIzol reagent (Invitrogen, Carlsbad, CA, USA) and NucleoSpin® RNA Clean-up kit (CapitalBio Company, China) according to the manufacturer’s instructions. The RNA quality was assessed by agarose-gel electrophoresis, and the RNA absorbance was measured by spectrophotometry. The ratio of the absorbance at 260/280 nm was then used to determine the RNA quality. 6 μg RNA of each sample was used for transcriptome sequencing. The RNA was processed for use on an RNA-seq platform (Illumina, Inc, San Diego, CA, USA) by the Chinese National Human Genome Center at Shanghai (CHGC).

mRNA [poly(A) RNA] was then purified from total RNA using Micropoly(A)Purist™ mRNA purification kit (Ambion, Cat.No.1919, Foster, CA, USA). The mRNA was fragmented and converted into a RNA-seq library using the mRNAseq library construction kit (Illumina Inc., San Diego, CA, USA) according to the manufacturer’s instructions. 2×100 bp paired-end sequencing was performed using the Illumina Genome Analyzer II x (Illumina GAIIx, San Diego, CA, USA).

### Sequence filtering and assembly

Sequence reads from all samples were cleaned using the FASTX toolkit (http://hannonlab.cshl.edu/fastx_toolkit/). First all the reads containing ‘N’ were discarded using a perl script, then adapter sequences were removed using the fastx_clipper program, followed by removal of quality < 5 bases from the 3′ end with fastq_quality_trimmer, requiring a minimum sequence length of 50 bp. Finally the reads with at least 90% bases > quality 20 were chosen using fastq quality filter for further assembly. *De novo* transcriptome assembly was performed using Trinity RNA-seq assembly v2013-02-25 with default parameters [PMID: 21572440].

Meanwhile, all sequence reads generated by Illumina sequencing were aligned to the reference transcriptome dataset using SOAP2 software [82].

### DEG identification

Based on the Trinity assembly results, the number of reads for each contig from each sample (control, defoliation, and grazing) was converted to reads per kilobase per million (RPKM) [83]. The MARS (MA-plot-based method with random-sampling model) module in the *DEGseq* package was used to calculate the differential expression of each contig between the analyzed samples [40]. The package *DEGseq* is a free R package for identifying DEGs from RNA-seq data. We used an FDR (false discovery rate) to determine the threshold p-value. An FDR < 0.001 was considered to indicate a significant difference in expression between the control and treated samples.

### Functional annotation, classification, and pathway analysis of DEGs

The sheepgrass transcriptome sequencing data had previously undergone Gene ontology (GO) annotation using a BLASTP search against the Swiss-Prot and TrEMBL databases with an E-value ≤ 1e-5 [84]. A GO functional classification was performed using WEGO software (http://wego.genomics.org.cn) to understand the distribution of gene functions in grazed sheepgrass [85].

In addition, Kyoto Encyclopedia of Genes and Genomes (KEGG) pathway annotation was performed using the KEGG Automatic Annotation Server (KAAS) with the bi-directional best-hit method. For KEGG, KAAS annotates every submitted sequence with a KEGG orthology (KO) identifier representing an orthologous group of genes directly linked to an object in the KEGG pathways and BRITE functional hierarchy [86, 87]. KEGG pathway enrichment analysis was conducted using KOBAS 2.0 (http://kobas.cbi.pku.edu.cn/, [88]).

Finally, EuKaryotic Orthologous Groups (KOG) annotation was carried out against the NCBI KOG database with a typical cut-off E-value ≤ 1e-5. The KOG annotations of the DEGs were classified into 25 protein functions and compared between the defoliation and grazing treatments. KOG enrichment analysis was conducted through hypergeometric distribution testing using the Phyper function in the R software package (http://www.rproject.org/). The Bonferroni correction was used to adjust the p-values. The significantly enriched functional clusters were selected based on a corrected q-value (< 0.05).

### DNA extraction and DNA-fragmentation assay

Using the same plant material, the cut ends of the leaves were collected every day for 9 days after clipping and daubing with water or BSA. Unclipped leaves were used as controls. Each sample for DNA extraction consisted of ten 1-cm-long leaf pieces. The leaves were flash-frozen in liquid nitrogen and stored at −80°C. The leaf tissues were ground to a fine powder in liquid nitrogen using a mortar and pestle, and the DNA was extracted using a plant genomic-DNA extraction kit (TIANGEN, Beijing, China) according to the manufacturer’s protocol. The total DNA was treated with RNase A (TaKaRa Bio Inc., Dalian, China) to remove any contaminating RNA. The isolated DNA, which was mainly derived from apoptotic cell bodies, was electrophoresed on a 2.0% agarose gel at 50 V for 3 h. The DNA fragments, which consisted of 160–200 bp multimers, were visualized under ultraviolet light after staining with ethidium bromide.

### Measurement of cell oxidative factors concentrations in the leaves

Using the same plant material, the leaves were clipped and daubed with distilled water, BSA solution or OVA solution. Three independent replicates were performed. Three days later, 1 cm of the cut end of each completely expanded leaf was collected, and approximately 30 cut ends were pooled. We chose the first complete leaf ends for further experiments. The corresponding parts of unclipped seedlings were collected as controls. The samples were frozen in liquid nitrogen and ground to a fine powder using a mortar and pestle. Approximately 0.2 g of each crude extract was added to 5 ml of pre-chilled PBS (50 mM at pH 7.8), thoroughly mixed, and centrifuged for 20 min at 4,000 g at 4°C. The supernatants were simultaneously assayed using the TBA (to measure MDA), KI (to measure H_2_O_2_), and NBT (to measure SOD) methods [89–91]. The absorbance values were measured using a 2600 UV spectrophotometer (UNICO, Shanghai, China).

### Leaf-scar measurements and phenotype comparisons

Using the same plant material, two-thirds of the first and last completely expanded leaves on each seedling were cut off at 10:00 AM. After clipping, the cut surfaces of the leaves were immediately daubed with water, 1 mM BSA or 1 mM OVA. Approximately 120 completely expanded leaves were removed per treatment. Three days later, the leaf scars were measured, and statistical analyses were performed to evaluate the effects of BSA on the first and last completely expanded leaves. Three independent replicates were performed. The statistical analyses were conducted using SAS 9.0 (SAS Institute, Cary, NC, USA) to compare the differences among the three treatments.

## Electronic supplementary material

Additional file 1: **Clean reads in seven sample libraries.** (JPEG 327 KB)

Additional file 2: **The quality of the assembly transcripts.** (JPEG 683 KB)

Additional file 3: **The analysis of sequencing saturation in seven samples.** The horizontal axis stands for the number of reads. The vertical axis stands for the contig obtained by contig assembly. (JPEG 315 KB)

Additional file 4: **The number of reads that contigs contain in seven samples.** (JPEG 718 KB)

Additional file 5: **The summary of WEGO output data of sheepgrass genes expressed in response to grazing stress.** (DOCX 14 KB)

Additional file 6: **The cluster analysis of differently expression genes among the control, defoliation and grazing treatments.** (PNG 100 KB)
