# Supplementary figures and images for: Bovine serum albumin in saliva mediates grazing response in Leymus chinensis revealed by RNA sequencing
Source: BMC Genomics. 2014 Dec 17;15(1):1126. doi: 10.1186/1471-2164-15-1126 (PMC4320431; doi:10.1186/1471-2164-15-1126)

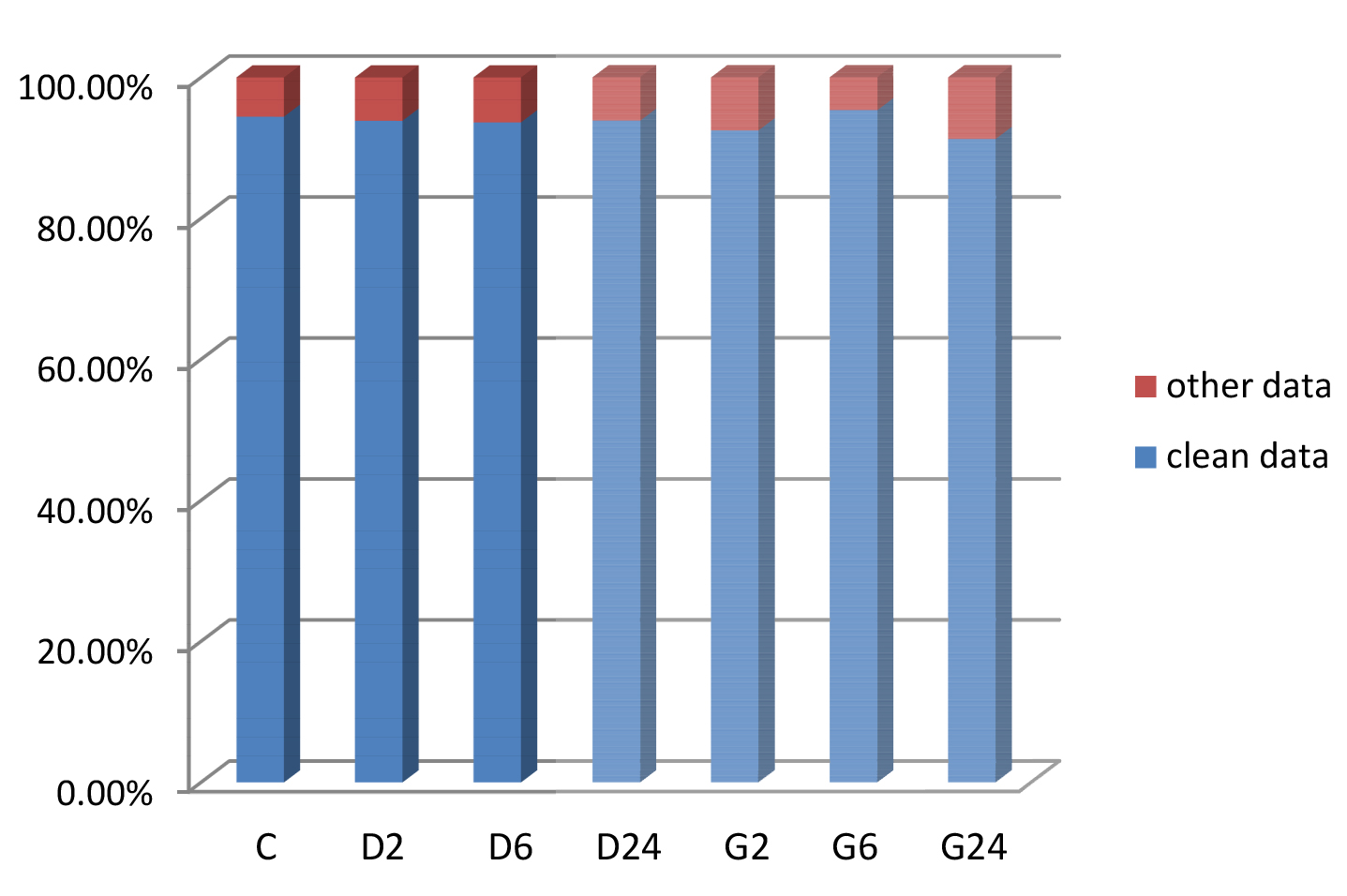

Supplement: Supplementary file 1 — Additional file 1: Clean reads in seven sample libraries. (JPEG 327 KB) [file 12864_2014_6921_MOESM1_ESM.jpeg]

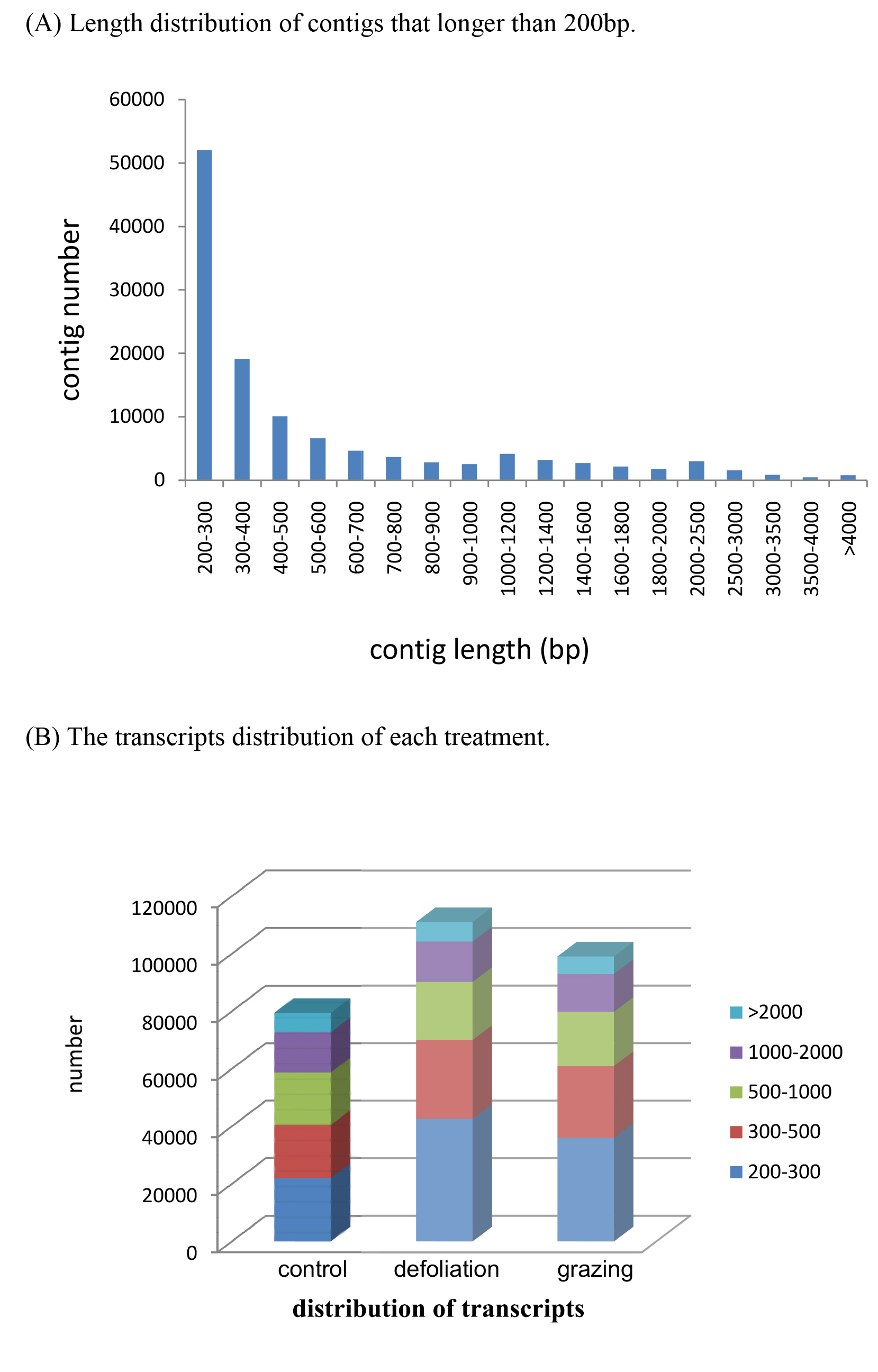

Supplement: Supplementary file 2 — Additional file 2: The quality of the assembly transcripts. (JPEG 683 KB) [file 12864_2014_6921_MOESM2_ESM.jpeg]

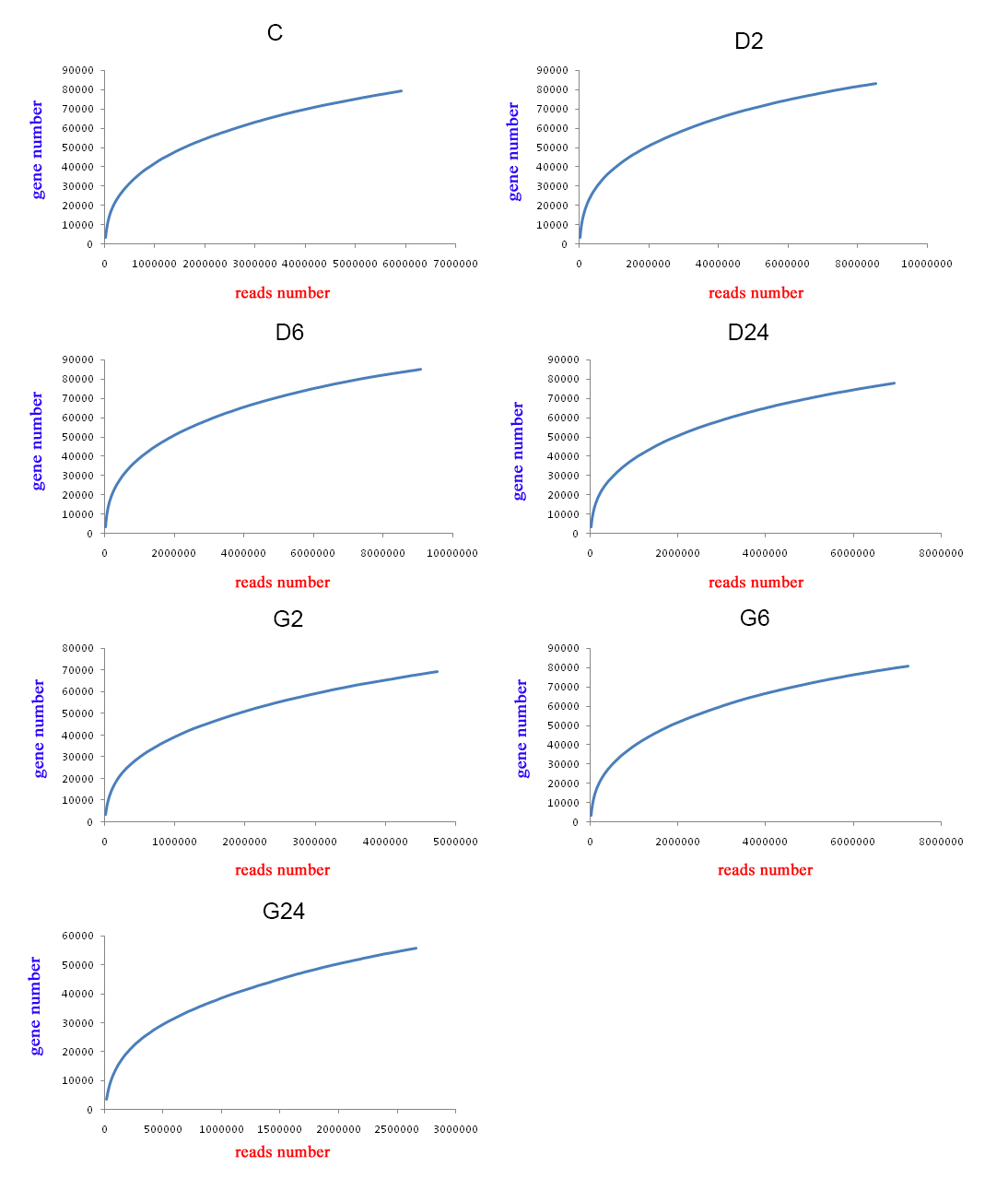

Supplement: Supplementary file 3 — Additional file 3: The analysis of sequencing saturation in seven samples. The horizontal axis stands for the number of reads. The vertical axis stands for the contig obtained by contig assembly. (JPEG 315 KB) [file 12864_2014_6921_MOESM3_ESM.jpeg]

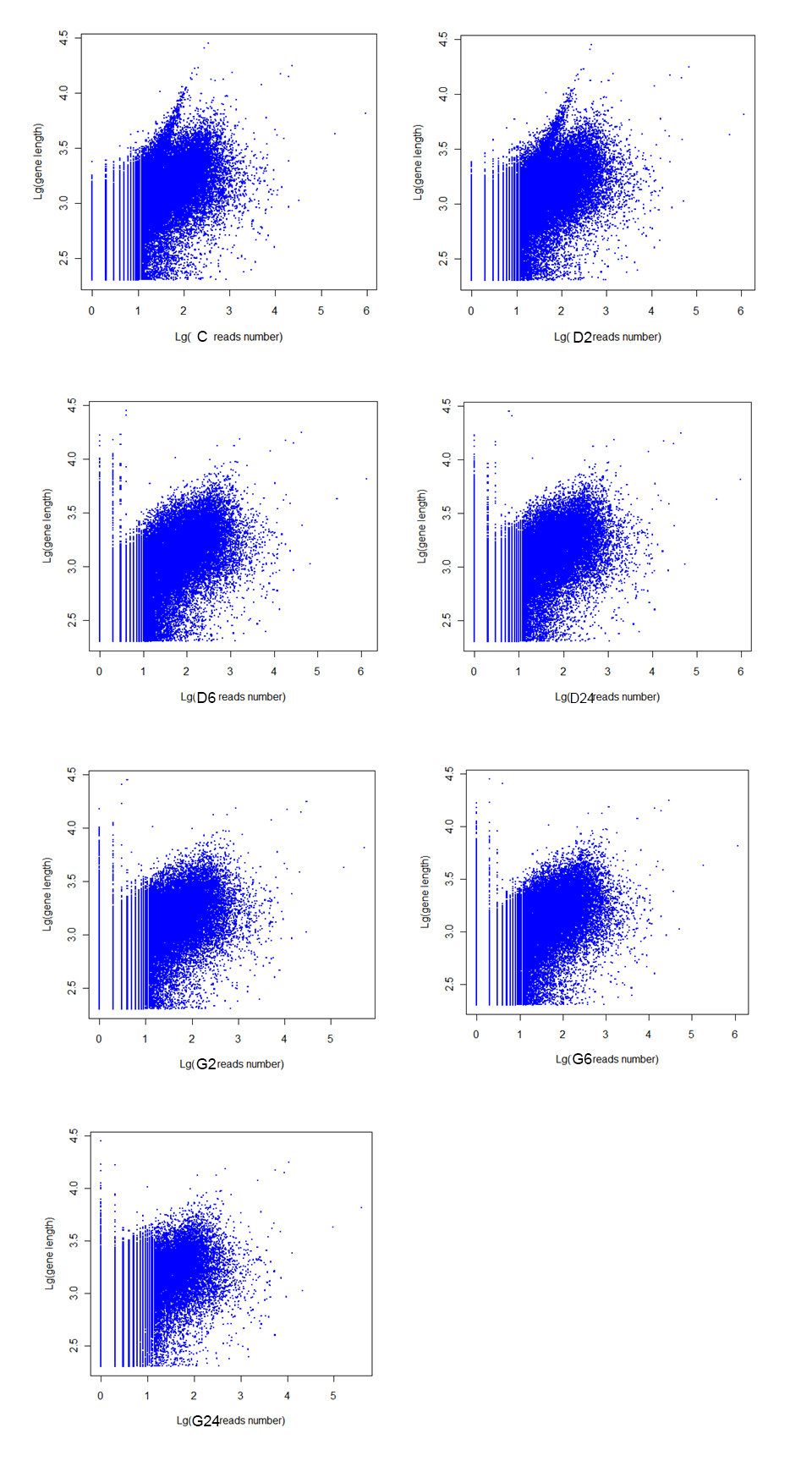

Supplement: Supplementary file 4 — Additional file 4: The number of reads that contigs contain in seven samples. (JPEG 718 KB) [file 12864_2014_6921_MOESM4_ESM.jpeg]

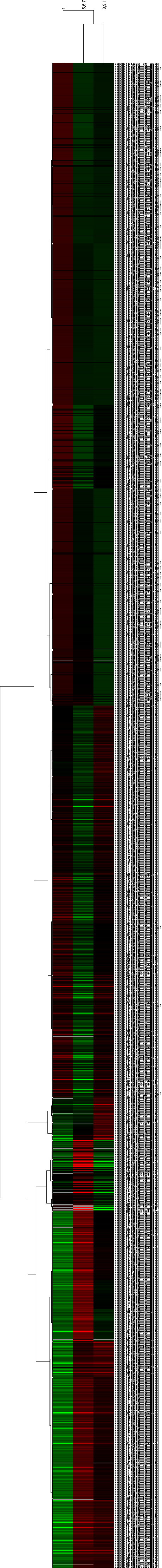

Supplement: Supplementary file 6 — Additional file 6: The cluster analysis of differently expression genes among the control, defoliation and grazing treatments. (PNG 100 KB) [file 12864_2014_6921_MOESM6_ESM.png]
