# Supplementary material for: Bovine serum albumin in saliva mediates grazing response in Leymus chinensis revealed by RNA sequencing
Source: BMC Genomics. 2014 Dec 17;15(1):1126. doi: 10.1186/1471-2164-15-1126 (PMC4320431; doi:10.1186/1471-2164-15-1126)
Supplement: Supplementary file 5 — Additional file 5: The summary of WEGO output data of sheepgrass genes expressed in response to grazing stress. (DOCX 14 KB) [file 12864_2014_6921_MOESM5_ESM.docx]

Additional file 5 – the summary of WEGO output data of sheepgrass genes expressed in response to grazing stress.

**Annotated transcripts number** **(Percentage)** **GO number** GO term

**Cellular Component:**
**420** **(4.3)** **GO:0005576** extracellular region
**42** **(0.4)** **GO:0044421** extracellular region part
**7508** **(76.4)** **GO:0005623** cell
**7508** **(76.4)** **GO:0044464** cell part
**323** **(3.3)** **GO:0031974** membrane-enclosed lumen
**401** **(4.1)** **GO:0031975** envelope
**830** **(8.4)** **GO:0032991** macromolecular complex
**4311** **(43.9)** **GO:0043226** organelle
**1643** **(16.7)** **GO:0044422** organelle part
**42** **(0.4)** **GO:0044421** extracellular region part
**1643** **(16.7)** **GO:0044422** organelle part
**2** **(0.0)** **GO:0044456** synapse part
**7508** **(76.4)** **GO:0044464** cell part
**2** **(0.0)** **GO:0045202** synapse
**2** **(0.0)** **GO:0044456** synapse part
**14** **(0.1)** **GO:0055044** symplast
------------------------------------
 **Total Terms:**16

**Biological Process:**
**254** **(2.6)** **GO:0044085** cellular component biogenesis
**903** **(9.2)** **GO:0032502** developmental process
**274** **(2.8)** **GO:0000003** reproduction
**653** **(6.6)** **GO:0016043** cellular component organization
**136** **(1.4)** **GO:0016265** death
**272** **(2.8)** **GO:0022414** reproductive process
**103** **(1.0)** **GO:0002376** immune system process
**1701** **(17.3)** **GO:0050896** response to stimulus
**968** **(9.8)** **GO:0032501** multicellular organismal process
**167** **(1.7)** **GO:0010926** anatomical structure formation
**172** **(1.7)** **GO:0051704** multi\-organism process
**1511** **(15.4)** **GO:0051234** establishment of localization
**11** **(0.1)** **GO:0022610** biological adhesion
**6378** **(64.9)** **GO:0008152** metabolic process
**1** **(0.0)** **GO:0016032** viral reproduction
**6** **(0.1)** **GO:0048511** rhythmic process
**1902** **(19.3)** **GO:0043473** pigmentation
**40** **(0.4)** **GO:0040011** locomotion
**1546** **(15.7)** **GO:0051179** localization
**90** **(0.9)** **GO:0040007** growth
**6690** **(68.1)** **GO:0009987** cellular process
**2011** **(20.5)** **GO:0065007** biological regulation
------------------------------------
**Total Terms:**22

**Molecular Function:**
**86** **(0.9)** **GO:0009055** electron carrier activity
**677** **(6.9)** **GO:0060089** molecular transducer activity
**534** **(5.4)** **GO:0030528** transcription regulator activity
**106** **(1.1)** **GO:0030234** enzyme regulator activity
**5927** **(60.3)** **GO:0003824** catalytic activity
**6850** **(69.7)** **GO:0005488** binding
**100** **(1.0)** **GO:0016209** antioxidant activity
**119** **(1.2)** **GO:0045182** translation regulator activity
**291** **(3.0)** **GO:0005198** structural molecule activity
**1** **(0.0)** **GO:0010860** proteasome regulator activity
**816** **(8.3)** **GO:0005215** transporter activity
------------------------------------
**Total:**11

========================================
**Total GO terms in three main categories:**49
